# 90% Accuracy for Photoplethysmography-Based Non-Invasive Blood Glucose Prediction by Deep Learning with Cohort Arrangement and Quarterly Measured HbA1c

**DOI:** 10.3390/s21237815

**Published:** 2021-11-24

**Authors:** Justin Chu, Wen-Tse Yang, Wei-Ru Lu, Yao-Ting Chang, Tung-Han Hsieh, Fu-Liang Yang

**Affiliations:** 1 Research Center for Applied Sciences, Academia Sinica, 128 Academia Rd., Sec. 2, Nankang, Taipei City 11529, Taiwan; nk95061313@gmail.com (J.C.); yp1369yang@gmail.com (W.-T.Y.); jackielu4119@gmail.com (W.-R.L.); thhsieh@gate.sinica.edu.tw (T.-H.H.); 2 Department of Biomechatronics Engineering, National Taiwan University, No. 1, Sec. 4, Roosevelt Rd., Taipei City 10607, Taiwan; 3 Division of Cardiology, Department of Internal Medicine, Taipei Tzu Chi Hospital, Buddhist Tzu Chi Medical Foundation, No. 289, Jianguo Rd., Xindian Dist., New Taipei City 23142, Taiwan; necrosparkeps@tzuchi.com.tw

**Keywords:** cohort, HbA1c, photoplethysmography (PPG), non-invasive, blood glucose, NIBG, deep learning

## Abstract

Previously published photoplethysmography-(PPG) based non-invasive blood glucose (NIBG) measurements have not yet been validated over 500 subjects. As illustrated in this work, we increased the number subjects recruited to 2538 and found that the prediction accuracy (the ratio in zone A of Clarke’s error grid) reduced to undesirable 60.6%. We suspect the low prediction accuracy induced by larger sample size might arise from the physiological diversity of subjects, and one possibility is that the diversity might originate from medication. Therefore, we split the subjects into two cohorts for deep learning: with and without medication (1682 and 856 recruited subjects, respectively). In comparison, the cohort training for subjects without any medication had approximately 30% higher prediction accuracy over the cohort training for those with medication. Furthermore, by adding quarterly (every 3 months) measured glycohemoglobin (HbA1c), we were able to significantly boost the prediction accuracy by approximately 10%. For subjects without medication, the best performing model with quarterly measured HbA1c achieved 94.3% prediction accuracy, RMSE of 12.4 mg/dL, MAE of 8.9 mg/dL, and MAPE of 0.08, which demonstrates a very promising solution for NIBG prediction via deep learning. Regarding subjects with medication, a personalized model could be a viable means of further investigation.

## 1. Introduction

An accurate and reliable non-invasive blood glucose (NIBG) measuring technique has long been in demand and extensively studied. While finger-prick blood glucose (BG) measurement causes pain and discomfort due to its invasive nature and introduces the risk of infection [1,2], NIBG technology can greatly improve the quality of life in diabetic patients by alleviating the pain of regularly invasive measurement [3,4,5,6,7,8]. Among the investigated NIBG measurements, photoplethysmography (PPG) has been long anticipated since this technique is simple, low-cost, already commonly implanted on various wearable devices [9,10,11,12,13,14,15], and has already been successfully applied to the measurement of oxygen saturation (SpO_2_) and pulsation rate. A PPG device measures the changes in transmittance or reflectance of near-infrared when blood passes through peripheral capillaries. It is known that the light absorption and reflectance of specific wavelengths are sensitive to the body’s hemodynamic properties, which are highly correlated to the health status of the cardiovascular system, and the cardiovascular system is influenced in the long term by BG levels that are directly measurable as pulse morphological profiles [9,11,13,16]. As a result, finding a correlation between PPG pulse morphology and BG levels could be a viable way towards NIBG prediction.

Previous studies on post analysis of NIBG measurements cover various different machine-learning models, such as support vector machine (SVM) [17], random forest [18], K-nearest neighbor (KNN) [19], Gaussian process regression (GPR) [20], and artificial neural network (ANN) [21]. Many morphological profiles and heart-rate-variance (HRV) features extracted from PPG signals are correlated to vascular function [22] and autonomic neuropathy [23] of the human body. Different signal-processing methods, such as fast Fourier transform (FFT), Kaiser-Teager energy (KTE), and spectral entropy were also exploited to extract features in different domains [9]. Some previous PPG-based NIBG studies in the past decade are summarized in Table 1. It is observed that even though these results were claimed to be promising with their limited number of testing subjects, none of them is yet successfully applied to commercial products for clinical usage. This suggests that the applicability of these approaches in a wide population of human beings might be quite limited.

Based on this observation, it is indicated that a larger data set might introduce more undocumented variations. A single universal model is not enough to cover all the cases and may lead to reduced accuracy. One of the major variations may originate in each subject’s ongoing medication, which may have a significant negative effect on prediction accuracy. For example, as PPG-based NIBG prediction highly relies on the correlation of BG level with the cardiovascular system, medical treatment of cardiovascular disease may alter the vascular status, and then further breaks the correlation of PPG to BG level. As a result, we came up with a hypothesis: the complex effects of different medications on human bodies may detach their actual physiological status from their presented PPG signal.

In this study, we arranged data of recruited subjects into cohorts with and without medication to verify our hypothesis with a large sample size of over two thousand subjects. We aimed to explore how the NIBG prediction discrepancy could be caused by the usually neglected effects of medications.

**Figure 1:**
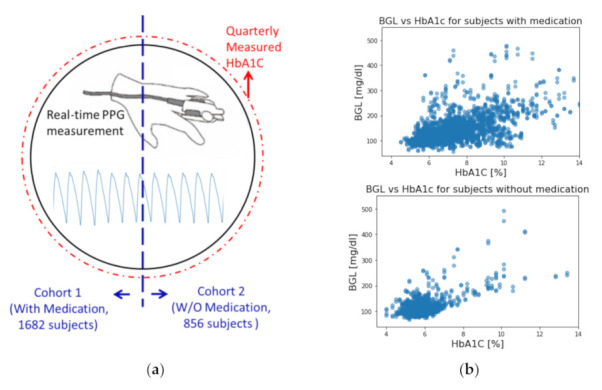
(**a**) Visual representations of this study. (**b**) Correlation of HbA1c and BG level for subjects with and without medication.

Furthermore, in order to improve our model prediction, we also incorporated quarterly measured glycated hemoglobin (HbA1c, sometimes referred to as A1C) as a feature in our training data. HbA1c is hemoglobin in the bloodstream that chemically bonds with glucose over time. It has been recognized by the American Diabetes Association as one of the powerhouse indexes for diagnosing diabetes and identifying prediabetes. Measurement of HbA1c levels can help to determine average BG levels and can be conducted at any time without prior dietary preparation (e.g., fasting). Unlike blood glucose tests, HbA1c is not affected by day-to-day variability. HbA1c values can be represented in either diabetes control and complication trial units (DCCT, %), or International Federation of Clinical Chemistry units (IFCC, mmol/mol). In this study, all HbA1c values are reported in the DCCT units with percentage (%). Measured HbA1c reflects the average blood glucose level of approximately the past three months due to the limited lifespan of red blood cells. The recommended cut-off value of HbA1c for diagnosing diabetes is at 6.5%. However, an HbA1c value less than 6.5% does not rule out the possibility of diabetes [24]. Although there exists a correlation between HbA1c and BG level, they are not directly interchangeable, as shown in Figure 1b. Since HbA1c only needs to be measured quarterly, which should not cause too much discomfort and inconvenience, acceptable for our model building.

## 2. Materials and Methods

In this study, a model that used all the 2538 subjects was established as the baseline to stand for the conventional methods of using a single universal model on a large sample size. We then separated subjects into two cohorts based on whether they were under medication of any sort (including insulin injections, cardiovascular treatments, etc.). The cohorts of subjects with and without medication consist of 1682 and 856 subjects, respectively. The characteristics of each cohort are summarized in Table 2. The first set of models was constructed for each cohort to compare with the universal model. The second set of models was then constructed for each cohort with the addition of their HbA1c values as a new feature to assess the performance improvement. All models use exactly the same CNN architecture for impartial comparison involving medication and HbA1c. A visual representation of the idea of this study is presented in Figure 1a.

### 2.1. Experimental Setup

The collection of samples in this study has been approved by the Institutional Review Board of Academia Sinica, Taiwan (Application No: AS-IRB01-16081). The samples were collected from a total of 2538 volunteer subjects. All subjects were fully informed and consented to the collection of data and their use. Two consecutive one-minute-long PPG signal segments were recorded for each subject with their physiological information such as age, body shape, blood pressure, HbA1c, BG level, and a questionnaire of whether the subject had any ongoing medical treatments. Medical treatments were classified into 4 categories, namely hyperglycemia, hypertension, hyperlipidemia, and other medical-doctor-prescribed medications (for example, cancer, sleeping pills, antidepressants, etc.). The detailed experimental setup and procedures can be found in [25].

Each minute-long PPG signal was first segmented into pulses for extracting both morphological features and heart-rate variance (HRV) features. Firstly, the valleys of the PPG waveform were identified using the Bigger-Fall-Side algorithm [26]. Then, from each valley, a backward one-second-long segment (total of 250 data points) containing the pulse was extracted. Averaging over the pulses, it was used to represent the entire minute of the PPG signal for deep-learning neural network.

In addition to the averaged PPG pulse waveform, the other features used in this study can be categorized into 3 categories, namely personal physiological features, pulse morphological features, and heart-rate variance. Personal physiological features include age, waist circumference, body mass index, and systolic and diastolic blood pressure. The pulse morphological features taken from the averaged PPG pulse contain the width of the pulse at 50% height, total pulse area of the minute, average of the pulse area, the median of the pulse area, and time difference from pulse valley to peak. The heart-rate-variance-related features taken from the segmented one-minute sample consist of both low-and high-frequency power from FFT, total power from FFT, percentage of pulse successive interval changes exceeding 20 ms, and the standard deviation of successive interval changes. The total of 17 features listed above (plus HbA1c for models including it) was aggregated into a feature vector, *F*, for feeding into each model.

### 2.2. Model Architecture

**Figure 2:**
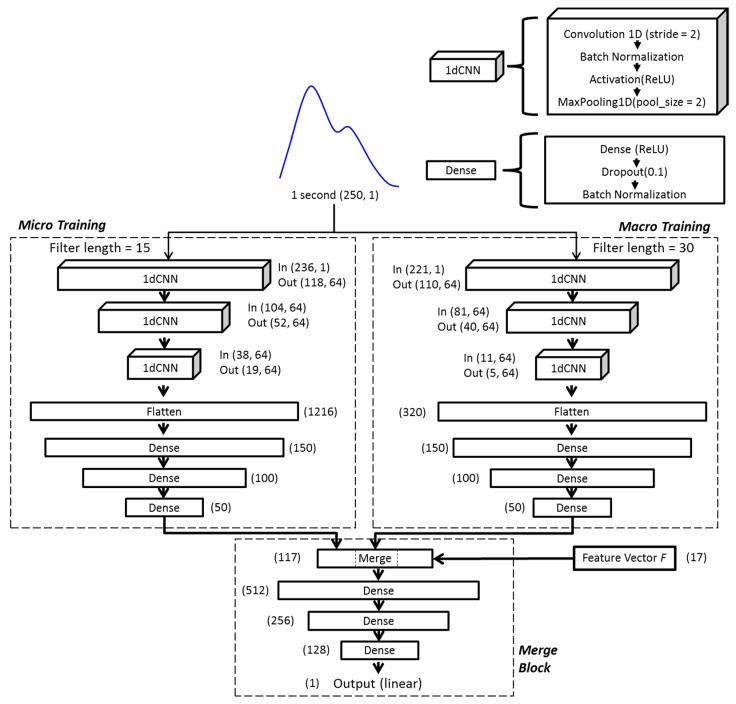
The architecture of the deep-learning model used in this study. The layers labeled ‘1dCNN’ are 1-dimension convolutional neural networks, and the layers labeled ‘Dense’ are fully connected neural networks. The numbers labeled in each layer are the shape (dimension and number of elements) of input/output data.

Inspired by object recognition with cortex-like mechanisms [27] and Google’s LeNet Inception structure [28], the model architecture used in this study comprises two parallel training blocks (micro training and macro training blocks) with different kernel sizes (filter lengths), followed by the merge block. The idea of this design is based on the theory of ventral stream of visual cortex [29,30], which aims to build a model of transformation invariance viewpoint for object recognition from input images. Briefly, considering the mechanisms of object recognition by human or mammals, objects in clutter can be easily recognized from viewpoints of any direction, orientation, and scale (i.e., short or long distances) to the object because our brains were trained in this way. As a result, the cortex-like model built a set of filters to capture object features from different viewpoints of direction, orientation, and scale via convolution and concatenated all the information to train the deep neural network. This complex hierarchy is imperative for 2D or 3D object recognition and could be quite computing demanded so that elaborated design and optimization are required. But in our problem, in which only the 1D feature extraction of PPG waveform is needed, such a complex hierarchy can be largely simplified. In our implementation, we only kept the feature extraction from viewpoints of different scales and restricted to two different scales with different filter lengths in the hopes of gathering both macro and micro views of the waveform of input data. With our preliminary tests, the combination of filter lengths of 15 for micro view and 30 for macro view showed the most promising prediction results, which led to our model design shown in Figure 2.

In each of the micro and macro training blocks, the same averaged one-second-long segment was taken and trained independently. Both training blocks consist of three consecutive one-dimensional CNN (1dCNN) layers, each followed by batch normalization and maximum pooling [31]. The results of 1dCNN layers were then flattened and fed into 3 fully connected layers in each block. The outputs of both training blocks were then combined with morphological features (feature vector F) before passing through the final merge block, consisting of three layers of fully connected neural network. Rectified linear units (ReLU) were used as activation functions throughout the model, except the linear function was used at the final output layer. The model used mean squared error as the loss function, which reads:

(1) $$\mathrm{MSE}\;=\;\frac{1}{N}\overset{N}{\underset{i\;=\;1}{\sum }}{\left(G_{i}\;-\;{\bar{G}}_{i}\right)}^{2}$$

where $G_{i}$ is the predicted BG value, ${\bar{G}}_{i}$ is the measured BG value (the ground truth), and N is the total number of samples. The schematic diagram of the model architecture is shown in Figure 2.

## 3. Results

**Figure 3:**
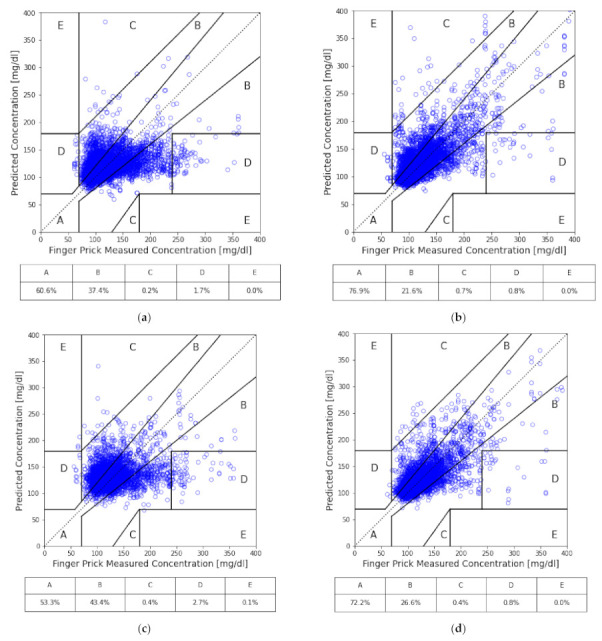

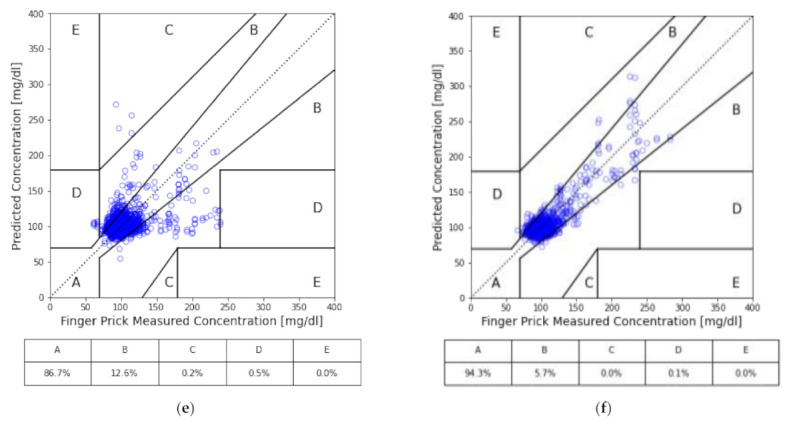
CEG analysis of our models: (**a**) all subjects; (**b**) all subjects with HbA1c as input; (**c**) the cohort of medication; (**d**) the cohort of medication with HbA1c as input; (**e**) the cohort of no medication; and (**f**) the cohort of no medication with HbA1c as input.

The universal model of not separating subjects into cohorts nor including HbA1c was established as a baseline to examine the effects of medication and the inclusion of HbA1c on the other models. In this study, the following metrics were used to assess the performance of models: root mean squared error (RMSE), mean absolute error (MAE), mean absolute percentage error (MAPE), coefficient of determination ($R^{2}$), proportion within 10% variance (±10%), and Zone A to Zone E of Clarke’s error grid (CEG) [32]. For each model, a total of 10 independent runs of training and testing were conducted with randomly separated data into 9:1 of training and testing sets to properly check the learning consistency and also to avoid overfitting. The average performance of the 10 independent trainings of each model is summarized in Table 3, while in Figure 3, the testing performance of all models (with the accumulation of all tests from the 10 trainings) is illustrated in CEG plots, which offer a quick and intuitive way of interpreting the estimated BG level. In CEG plots, all the estimated results are classified into 5 zones (A to E) based on how well the estimation affects clinical decision making, among which zone A is considered accurate, zone B is considered clinically acceptable (does not lead to negative impacts), and zones C, D and E are considered dangerous due to significant clinical error in predictions.

By separating subjects into two cohorts based on whether they are under any medication, we can see the performance increased in the cohort without medication. On the contrary, the performance got worse in the cohort with medication. Compared to the baseline, the cohort of subjects with medication, while excluding HbA1c, had predictions of approximately 7% less in Zone A of CEG; while for the cohort of subjects without medication nor HbA1c, it significantly improved by around 26%. Similar behavior was found across all the other performance metrics for both cohorts.

Furthermore, with the inclusion of HbA1c, each case gained a significant improvement in their overall performance. The prediction accuracy of the model with HbA1c in the cohort with medication improved from 53.3% to 72.2% of predictions in zone A of CEG. Interestingly, although improved by adding HbA1c, the baseline universal model only gained 16% improvement, up to about 77% of predictions in zone A of CEG, while the cohort with medication just barely outperformed the baseline universal model without HbA1c. Both are still undesirable in practical application. On the other hand, the performance of the model with HbA1c in the cohort without medication achieved 94% of predictions in zone A of CEG and had no prediction fall into zones C, D or E in testing sets.

As one may argue that the poor performance of the model of the cohort with medication may be the result of more samples (about twice the amount compared to the cohort without medication) leading to larger deviation, we provide two tests showing that it is not the case. Firstly, by examining MAPE in Table 3, which is a measure of averaged predicting deviation in normalized BG level, defined as:

(2) $$\mathrm{MAPE}\;=\;\frac{1}{N}\overset{N}{\underset{i\;=\;1}{\sum }}\left|\frac{{\bar{G}}_{i}\;-\;G_{i}}{{\bar{G}}_{i}}\right|$$

**Figure 4:**
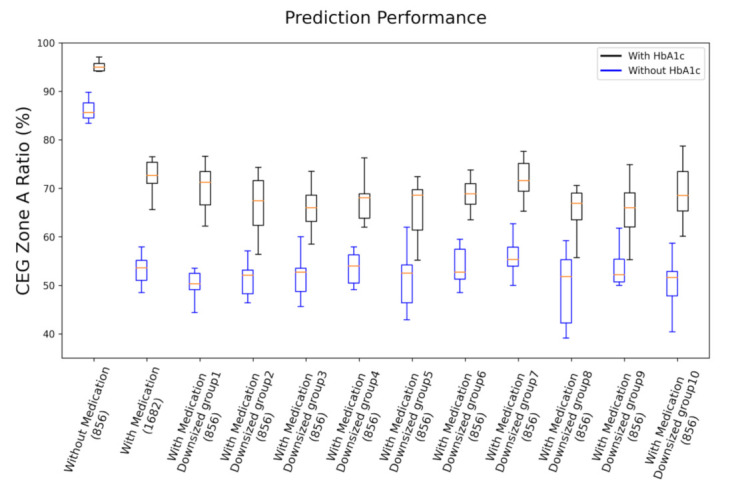
Performance comparison of cohorts without medication (856 samples), with medication (1682 samples), and with medication and downsized samples (randomly selected 856 samples, 10 replications) in ratio of zone A of CEG plot. Both results of with HbA1c and without HbA1c are presented.

We see that it is approximately 12% higher for the cohort with medication than the cohort without medication. This indicates that overall, the cohort with medication had larger relative deviation to the ground truth. Secondly, in Figure 4, we present the performance comparison of cohorts without medication (856 samples), with medication (1682 samples), and with medication and downsized samples (randomly selected 856 samples from the total 1682 samples) in ratio of zone A of CEG plot. The tests of downsized cohorts with medication were repeated 10 times; each time, the test followed the same procedure of 10 repeats of 9:1 data splitting for training and testing, as described previously. Thus, results of the 10 repeats in each case are summarized in the box plot. As shown in Figure 4, all the cohorts with medication reveal roughly the same prediction accuracy, which is significantly lower than that of the cohort without medication, independent of the number of samples involved. This evidence suggests that medical treatment of subjects may be the source the degraded prediction capability for PPG-based NIBG. This confirms our suspicion that medication indeed has a negative effect on model performance.

**Figure 5:**
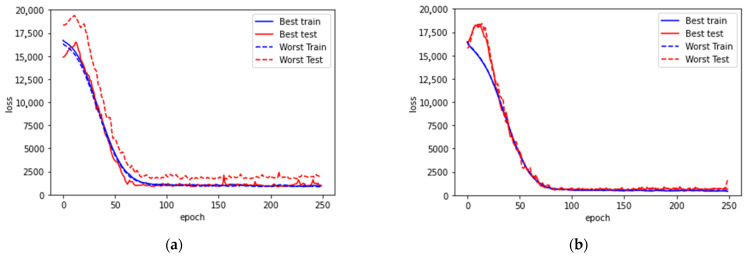

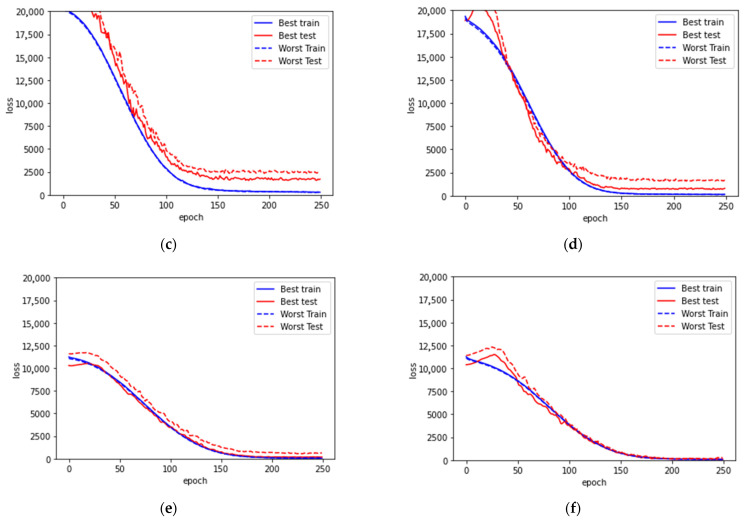
Learning curves for (**a**) model with all subjects and without HbA1c, (**b**) model with all subjects and with HbA1c, (**c**) model with medication, (**d**) model with medication and with HbA1c, (**e**) model without medication, (**f**) model without medication and with HbA1c. In each case, the best and worst trainings among the 10 trainings are presented. Both the best and the worst testing cases among the 10 trainings in each model are presented.

In Table 4, we list the average training and testing loss (Equation (1)) of the 10 trainings for each case. From the difference between the training and testing loss, we can quantify and objectively see the discrepancy between training and testing. In an ideal situation, one would like to minimize the loss during training and have the testing loss be as close to the training loss as possible. That would make sure that the model actually learned the pattern of data and led to accurate predictions for the testing set. The learning curves of our models are presented in Figure 5.

For the universal model with all subjects included, the loss on both training and testing are large. As shown in Figure 5a,b, the learning curve flattens out after 75 epochs, the shortest among all the cases. This suggests that compared to the other cases, there is conflicting information between the ground truth and the data patterns that forbids further convergence during learning.

From the discrepancy summarized in Table 4, the cohort of subjects with medication has significantly larger differences between their training and testing loss. From the learning curve (Figure 5c,d), although the loss converged much better during training, the resulting models cannot reproduce good predictions. In addition, the curves of testing loss are flattened after 125 epochs without bending up, which suggests that there is no overfitting. As a result, it is clear that the model is unable to learn properly for accurate estimations. There is some key information missing for further in-depth analysis of the cohort with medication.

On the contrary, the cohort of subjects without medication has very minor differences between their training and testing loss, especially with the inclusion of HbA1c. This further suggests that the key piece for improving prediction performance relies on mitigating the influence of medication.

## 4. Discussion

PPG morphological features extracted from the CNN architecture help to explain the dynamic glucose variance among the samples. However, the PPG waveform variation may also be influenced by a lot of factors, such as finger temperature, probe contact, blood pressure, etc. A key step to improve NIBG modeling is to find a basis for the model to distinguish the static part of glucose-related features. In this work, we took two factors into account: medication and the HbA1c level.

The result shows that excluding the medication-treated samples and including HbA1c as a feature improves prediction performance. The worse result of subjects under treatment implies that when there is medication involved, the problem becomes more complicated. As glucose homeostasis of a human body controls metabolism stability, external drug treatment may alter the body’s physiopathology, which, in turn, leads to confounded biological signal presentation, making the physiological status unpredictable. Even more, various kinds of medication and different dosages of drugs all result in complex effects. Here, we propose two possible solutions for mitigating the effects of varying medication between subjects. (1) Properly clarify the effects of treatments on our investigating target, the PPG signal representation to BG level prediction, and incorporate them into model building. This study requires more comprehensive data on the medication routine for each subject and in-depth knowledge of the biological influence of these treatments. (2) Design a personalized model for each subject. Both approaches have been demonstrated in the work of Weixi Gu et al. [33], though not using PPG. Particularly, a personalized model essentially detours all interpersonal differences, no matter whether medication is applied, and more specifically, targets the physiological evolution of oneself. This study requires long-term collection of personal data but could be a viable means of future investigation.

HbA1c plays an important role in improving our models. HbA1c has been regarded as a readily available measurement and is an important homeostasis index representing a long-term body glucose balance. HbA1c also reflects a portion of glucose persistent in the bloodstream that may have a certain influence on near-infrared light absorption. Practically, patients only need to measure their HbA1c once every three months, which is a significant advancement for people needing regular BG level monitoring. Therefore, it is feasible to adopt HbA1c for NIBG modeling.

One limitation in this study would be the data-imbalance issue. When separating the subjects into cohorts of with and without medication, this issue may become severe for subjects with high and jumbled glucose values, which may lead to significant outliers in prediction. This might not be easily resolvable since it depends on the recruited subjects available in our study. In order to prevent biased prediction due to unbalanced data, data reweighting or data augmentation to enhance training could be considered to evaluate the model.

Finally, for clinical applications, although our PPG-based NIBG method is currently in the preliminary stage and may require more improvement in various clinical settings, it could still be of benefit to some specific scenarios of treatments. For example, our method is readily applicable to some aspects of preventive healthcare, such as regular BG monitoring to control its progression for healthy or high-risk populations who are not under medical treatment yet but may suffer from severe diabetes-related problems. In addition, if the method further improved its prediction accuracy, it could also be competitive with continuous glucose monitoring (CGM) devices. Many CGM methods have been commercialized and are profoundly less invasiveness than the standard finger-prick sampling method. However, CGM sensors still require sampling of body fluids, such as sweat, interstitial fluids, tear, saliva, etc. [34]. Thus, CGM sensors have limited lifespan (normally between 7 and 14 days) and also have to be calibrated intermittently (every 12 to 24 hours) due to the potential 10–15% difference compared with finger-prick blood glucose. On the other hand, our method would further reduce invasiveness and extend monitor longevity by embedding a wearable device. Furthermore, with the input of HbA1c, our method could sustain high BG prediction accuracy without frequent calibration.

## 5. Conclusions

By analyzing model-prediction accuracies and learning curves, we demonstrated that a single universal model for NIBG prediction could suffer from uncontrollable factors, such as medication. By separating subjects into cohorts, the model with subjects without medication of any sort has approximately 30% higher prediction accuracy than its counterpart. Furthermore, adding HbA1c leads to this model achieving RMSE of 12.4 mg/dL, MAE of 8.9 mg/dL, MAPE of 0.08, and 94.3% prediction accuracy with no failed predictions falling into erroneous zones C, D and E of the CEG plot. We believe that the proposed model with cohort data arrangement and quarterly measured HbA1c is very promising for clinical usage for the population not taking any medication.

Nevertheless, we also found medications have significant impacts on NIBG estimation by altering one’s exhibited physiological signal and resulting in deviated predictions. With the input of HbA1c, we have done our best to boost the prediction accuracy of the cohort with medication by around 20% improvement in zone A of the CEG plot. For further improvement, we recommend the approach of personalized models to nullify diversities between individuals in order to achieve clinically acceptable performance. We believe it would be a more preferable solution to extend NIBG with machine learning to all populations.

## Figures and Tables

**Table 1:** Comparison of PPG-based NIBG predictions in the past decade with the present work.

Author (Year)	Number of Subjects	Prediction Accuracy (Ratio in Zone A of CEG)	Subjects Splitting for Modeling	Input Data	Method	Age of Population
E. Monte-Moreno [9] (2011)	410 subjects (79 diabetes)	87.71%	Universal model (not split)	Kaiser-Teager energy, spectral entropy, fast Fourier transform, energy profile, age, gender, BMI, SpO_2_, HR	Linear regression, support vector machine, random Forest, neural network	Age 9 to 80 (mean ± SD = 37.97 ± 13.32)
P. Jain et al. [16] (2019)	190 (80 healthy, 58 diabetics, 52 prediabetics)	94%	Universal model (not split)	Three-channel of PPG voltage value	Deep neural network	Age 17 to 77 (mean ± SD n/a)
S. Ramasahayam [12] (2015)	55 subjects	95.38%	Universal model (not split)	Optical densities	FPGA implementation of ANN	n/a
J. Yadav et al. [21] (2012)	50 normal subjects	86.01%	Universal model (not split)	Kaiser-Teager energy, spectral entropy, hr, person-specific information, galvanic skin response, skin temperature	Multi linear regression, artificial neural network	Age 21 to 30 (mean ± SD = 24 ± 3)
V. P. Rachim et al. [13] (2019)	12 healthy subjects	100%	Personalized model	24 features extracted from PPG (optical density, Kaiser-Teager, pulsatile component)	Linear partial least squares regression	n/a
R. Bunescu et al. [17] (2013)	10 subjects with type 1 diabetes	19.5 RMSE (on BGL 30 min in the future)	Universal model (not split)	Meal absorption dynamics, insulin dynamics, glucose dynamics, ARIMA generated feature	Support vector machine	n/a
**This Work**	2538 (1682 with medication, 856 w/o medication)	60.6–94.3%	PPG data with cohort arrangement (with and w/o medication)	Fast Fourier transform, pulse morphological, physiological, age	One-dimensional CNN with micro and macro training	Age 38 to 80 (mean ± SD = 63.15 ± 9.67)

**Table 2:** Characteristics of the participants of cohorts with and without medications.

	Cohort	BG (mg/dL, Mean ± SD)	HbA1c (%, Mean ± SD)	Age (Years, Mean ± SD)	BMI (kg/m^2^)	W_cir * (cm, Mean ± SD)
**Total of 2538 Subjects**	Subjects with medication (1682 subjects)	136.1 ± 43.6	7.3 ± 1.5	65 ± 9	25 ± 4.1	86.2 ± 10.2
	Subjects w/o medication (856 subjects)	103.3 ± 22.0	5.9 ± 0.8	59 ± 10	23.6 ± 3.5	80.3 ± 9.6

* W_cir: waist circumference.

**Table 3:** Summary of model performance.

Data Set	Subject Count	CEG Zone A (%)	RMSE (mg/dL)	MAE (mg/dL)	MAPE (%)	$R^{2}$	±10%
All (No HbA1c)	2538	60.6	36.7	25.4	19	0.06	0.33
All (with HbA1c)	2538	76.9	30.5	18.9	15	0.42	0.50
with Medication (No HbA1c)	1682	53.3	44.4	31.9	23	−0.09	0.28
with Medication (with HbA1c)	1682	72.2	32.1	21.7	16	0.39	0.43
w/o Medication (No HbA1c)	856	86.6	19.7	11.8	11	−0.05	0.6
w/o Medication (with HbA1c)	856	94.2	12.4	8.9	8	0.71	0.6

**Table 4:** The average training and testing loss (Equation (1)) of each model.

	Training Loss	Testing Loss	Difference (Test-Train)
All (No HbA1c)	884	1534	650
All (with HbA1c)	442	950	508
with medication (No HbA1c)	292	2176	1884
with medication (with HbA1c)	130	1052	922
w/o medication (No HbA1c)	57	485	428
w/o medication (with HbA1c)	75	165	90

## Data Availability

The data presented in this study are available on request to the corresponding author, but has to follow the aforementioned IRB guidelines.
